# Involving a black woman patient and public involvement group in maternal health methodology research: learning from the PROMISE Study

**DOI:** 10.1186/s40900-026-00983-5

**Published:** 2026-09-23

**Authors:** Sophie S. Hall, Georgia Clancy, Shireen Patel, Bhuvan Majmudar, Fatou Benga, Makini Jones, Catherine Henshall

**Affiliations:** 1 Nottingham Clinical Trials Unit, School of Medicine, University of Nottingham, Nottingham, NG7 2RD UK; 2 University of Nottingham, Jubilee Campus, Wollaton Road, Nottingham, NG8 1BB UK; 3 Egality Health Ltd., London, SW16 2LP UK; 4 University of Nottingham, Patient and Public Involvement (PPI) Member, Nottingham, England; 5 Oxford School of Nursing and Midwifery, Oxford Brookes University, Jack Straw’s Lane, Oxford, OX3 0BP UK

**Keywords:** Patient and public involvement, PPI, GRIPP2, Maternal health, Black women, Inclusive research

## Abstract

**Background:**

Patient and public involvement (PPI) is at the centre of high-quality health research, but PPI representatives are often limited in terms of demographic diversity and are often absent from methodology research. In maternity research, this exclusion is consequential, as Black women experience significant healthcare inequities, are often excluded from research, and yet are rarely involved in shaping how research is designed and conducted.

**Aim:**

To critically reflect with our PPI members on the development, processes, and impacts of PPI in the methodology study – PROMISE, which aimed to support Research Delivery Teams to engage Black women in discussions about opportunities to participate in maternal health research.

**Methods:**

PROMISE embedded PPI across the research lifecycle, including grant development, recruitment strategy design, data interpretation, and the co-design of outputs. A purposively recruited national PPI group of Black women with lived experience of pregnancy worked in partnership with researchers and a community engagement organisation. Together with PPI representatives, we drew upon the GRIPP2 long-form framework to articulate learning around conducting meaningful, inclusive PPI in maternal health and methodology research with Black women.

**Results:**

PPI shaped the study focus, challenged early assumptions about engagement, strengthened interpretation of qualitative findings, and helped co-design the main outputs of the PROMISE study, which included Principles of Engagement for supporting research delivery teams to engage Black women in maternity research. Through this reflective piece we co-developed lessons for conducting inclusive PPI with Black women, highlighting the importance of trust-building, cultural specificity, flexibility, emotional safety, and a recognition of the importance of interpreting findings through lived experience.

**Conclusions:**

Conceptually, the study positions inclusive PPI with Black women as a methodological necessity that reshapes research design, interpretation, and implementation. Alongside our reflections supported by the GRIPP2 framework, we describe practical approaches to implementing inclusive PPI processes in methodology research.

**Trial registration:**

Not applicable.

**Supplementary Information:**

The online version contains supplementary material available at https://doi.org/10.1186/s40900-026-00983-5.

## Abstract (plain language summary)

Patient and public involvement (PPI) means working with people who have real-life experience to help design and improve research. This is important because research should reflect the needs of the people it is trying to help. However, not everyone is included. Black women are often left out, even though they are more likely to have poorer experiences of healthcare, particularly during pregnancy. This study looks at how we involved Black women in a research project called PROMISE. This project was not just about health—it was about improving how research itself is done (this is called “methodology” research). This matters because if research is designed in the wrong way, it can exclude people before the study even begins. We worked closely with a group of Black women who had been pregnant. They helped shape the study from the start, helped us understand what the results really meant, and helped create guidance for researchers. Their ideas changed how we thought about the research and made sure it reflected real-life experiences. We learned that good involvement takes time, trust, and respect. It helps to work with trusted community groups, be flexible, and create safe spaces where people feel comfortable sharing their experiences. We show that involving Black women is important not only for better healthcare, but also for improving how research is designed and carried out. By including their voices in methodology research, we can make research fairer from the very beginning and ensure it works better for everyone.

**Supplementary Information:**

The online version contains supplementary material available at https://doi.org/10.1186/s40900-026-00983-5.

## Background

Patient and public involvement (PPI) is increasingly, and credibly, positioned as a core component of high-quality healthcare research [1–4]. This is underpinned by mandates from major national and international research funders (e.g., National Institute for Health and Care Research [NIHR], Wellcome) for patients and the public to be meaningfully involved across the research lifecycle as a condition of funding. PPI helps improve the relevance, acceptability, and impact of research. However, growing evidence suggests that participation in PPI activities often lacks diversity in terms of demographic characteristics, with contributors frequently drawn from relatively privileged groups who have the time, confidence, and familiarity to engage with academic research structures [5, 6]. Furthermore, PPI in methodology research (exploration/evaluation to improve the quality and efficiency of research) is often scarce, potentially contributing to the under-representation of key groups in research and undermining the quality of the health and medical research generated [7, 8]. Moreover, the lack of PPI in methodology research may be a contributing factor as to why methodology research experiences significant challenges when it comes to being implemented [9–11].

A lack of diversity in PPI is perhaps particularly consequential in maternity research and related methodology research. In the United Kingdom, Black women experience significantly poorer outcomes during pregnancy and childbirth [12, 13], yet their voices and lived experiences remain under-represented in research design, governance, and interpretation [14, 15]. When involvement does occur, it is often framed within broad and homogenising categories of “minoritised ethnic groups”, obscuring the specific structural, cultural, and historical factors shaping Black women’s experiences of healthcare and research [16–18]. Without the meaningful involvement of Black women in methodology research, decisions about how research is designed and conducted risks reproducing the very assumptions and blind spots that contribute to persistent inequities in maternity care. In this context, involving Black women is a methodological necessity to ensure that research supports the production of healthcare innovations that have been designed and evaluated by the people that need them the most.

There is a growing body of guidance to support the delivery of inclusive research and patient and public involvement, including the NIHR Innovations in Clinical Trial Design and Delivery for the Under-served (INCLUDE) framework, guidance from Trial Forge and Step Up [19–22], and established standards for the development, delivery, and reporting of PPI [23]. This guidance provides important high-level principles and has helped raise expectations around inclusivity and involvement in health research. However, translating these principles into practice remains challenging. Researchers at all stages of their careers report challenges in delivering inclusive and meaningful PPI, particularly in the context of competing time and funding pressures [1, 2].

Recruiting inclusive PPI user groups is further constrained by structural barriers such as limited time and financial resources for contributors, as well as limited awareness of how to get involved. These barriers are not experienced equally and may disproportionately affect individuals and communities from minoritised experiencing structural inequities. Despite an increasing emphasis on lived-experience involvement in research, the existing evidence highlights a persistent lack of diversity in the demographic profiles of PPI representatives [3, 24]. Moreover, while existing frameworks articulate *what* inclusive research should aim to achieve, they often provide limited operational guidance on *how* to work equitably with communities who may have experienced systemic racism, discrimination, or harm within healthcare and research systems. In this context, conventional PPI models risk tokenistic or extractive forms of engagement [25, 26], particularly where involvement is introduced late in the research process or resourced inadequately.

The aim of the PROMISE study (Principles of Engagement: Developing Methods to Increase Representation of Black Mothers in Maternal and Neonatal Healthcare Research) was to develop Principles of Engagement to support Research Delivery Teams (e.g. research nurses and midwives) discuss opportunities to participate in maternal and neonatal healthcare research with Black women. A large systematic review of the literatures [27], focus groups and consensus workshops [28], were conducted to uncover the cultural, environmental, and communication processes that shape Black women’s engagement with maternity and neonatal research. PROMISE was designed on the premise that PPI was methodologically necessary, not optional. Rather than treating ethnicity as a recruitment characteristic, PROMISE conceptualised Black women’s involvement as central to understanding how trust, communication, and participation in research were shaped by lived experience. Within PROMISE, the term Black women was not used to imply a homogeneous ethnic or cultural group. Rather, it was used as a socially and politically relevant category reflecting women who may share experiences of racialisation, marginalisation, and inequitable treatment within healthcare and research systems, while recognising substantial diversity in ethnicity, nationality, migration history, religion, language, socioeconomic circumstances, and individual experiences. This perspective informed our decision to focus specifically on Black women’s maternity and research experiences whilst avoiding assumptions of shared identities or viewpoints.

The Guidance for Reporting Involvement of Patients and the Public (version 2) (GRIPP2) is primarily intended as a reporting framework [23]. GRIPP2 is an internationally recognised reporting framework designed to improve the quality, transparency, and consistency of PPI reporting in health research. Here, we used the GRIPP2 long-form not as a prescriptive checklist, but as a framework to examine how involvement was operationalised in practice during PROMISE, what impact it had, and where limitations and tensions remained. This approach allowed us to move beyond descriptive reporting and towards a critical examination of relational and context-specific aspects of PPI with Black women, which are often under-explored in conventional accounts. We also recognise that GRIPP2 was not designed to fully capture the ethical and structural dimensions of working with communities who have experienced historical exclusion from research; these tensions were therefore made explicit in our reflections rather than treated as methodological shortcomings.

The aim of this paper was to use the GRIPP2 long-form framework to critically reflect on PPI within the PROMISE study. We focus on what was done, what impact it had, and what was learnt about conducting PPI with Black women in methodology research in the field of maternal health. In addition to reflecting on impact and learning, this paper provides practical insights into how inclusive PPI with Black women was operationalised, including recruitment strategies, reimbursement, facilitation approaches, relationship-building activities, and shared decision-making processes. The paper was co-led with two PPI members (FB, MJ), with their reflections integrated throughout the paper’s Findings and the Discussion sections.

## Methods

### Design of PPI

PPI plans were developed by the study team who had previous experience and training in PPI, alongside a leading community engagement organisation (Egality Health) who have expertise in improving exclusion in research. PPI was embedded across the lifespan of the PROMISE study, from informing the research objectives and methods to dissemination.

Egality Health were an important mechanism to support culturally appropriate engagement and to develop links with trusted community organisations. Their input began before funding was secured, which helped shape a realistic and proportionate approach to early PPI involvement in the context of limited time and financial resources. This early engagement supported clear thinking about what was feasible, avoidable burdens on community members, and how involvement could be meaningful rather than tokenistic. Egality Health helped design the study materials and connected the team with trusted community groups (such as the Black Women’s Group, Black Mamas Birth Village, Black Health Initiative, Research Black, the Caribbean African Health Network, and City of Sanctuary UK). These groups helped ensure Black women’s voices were included and supported recruitment of women of pregnancy age and played a key role by acting as trusted intermediaries (“facilitators”) to access communities experiencing structural inequities.

### Researcher positionality and reflexivity

The core academic research team was comprised of researchers who did not identify as Black women. We recognised that our social, professional, and institutional positions influenced how the research was designed, facilitated, interpreted, and reported. Throughout the study, we sought to approach involvement reflexively by openly acknowledging this positionality, inviting challenge from contributors, discussing power dynamics explicitly during meetings, and treating experiential knowledge as a form of expertise rather than validation of researcher interpretations. Reflexive discussions informed decisions about study materials, interpretation of findings, and the development of the Principles of Engagement guidance. In addition, we actively sought input from our study advisory group and PPI members who were Black women to shape the entire research process from design to data collect, interpretation and dissemination. While these efforts did not remove structural power imbalances, they helped make these dynamics visible and open to discussion throughout PROMISE. Cultural humility was operationalised through openness to learning from contributors, acknowledgement of the limits of researcher knowledge and experience, active reflection on assumptions, and willingness to adapt research decisions in response to feedback.

### The PPI group

Contributors were recruited from qualitative focus groups conducted as part of the PROMISE study. Eligible contributors were Black women aged 18 years or older with lived experience of pregnancy who had participated in PROMISE focus groups and expressed willingness to engage in ongoing involvement activities. We purposively sampled women to maximise diversity according to ethnicity, migration history, age, and maternity experiences whilst supporting continuity between data collection and subsequent involvement activities. To be eligible to join the group, members were required to self-identify as being a Black woman. Recruiting contributors this way supported continuity and enabled more confident, meaningful engagement. Twelve women were invited (via email) to join the PROMISE PPI group. Eleven of the twelve Black women contacted expressed interest, with the final PROMISE PPI group comprising 11 Black women aged 18 years or older. Contributors reflected diversity across Black African, Black Caribbean, and Black British identities, first- and second-generation status, and a range of educational and socioeconomic backgrounds (Table 1). Five of the 11 PPI members had taken part in research previously.

**Table 1:** PPI demographics

Demographic characteristics	(n=11)	%
Age (mean)	41.75 years	100
Country of birth:		
Nigeria	1	9
Sudan	2	18.1
Trinidad	1	9
United Kingdom	4	36.3
Prefer not to say	3	27.2
Sex:		
Female	11	100
Ethnicity:		
Black African	6	54.5
Black British	1	9
Black Caribbean	2	18.1
Prefer not to say	1	9
Other: Black British-Caribbean	1	9
Number of children:		
1	3	27.2
2	3	27.2
3	2	18.1
4	2	18.1
Prefer not to say	1	9
Have you ever taken part in a research study as a participant?		
Yes	5	45.4
No	2	18.1
Prefer not to say	4	36.3
Marital status:		
Married	8	72.7
Single	2	18.1
Prefer not to say	1	9
Highest qualification:		
Higher degree	4	36.3
Other higher qualification	1	9
First degree	3	27.2
A-level	1	9
No qualifications	1	9
Prefer not to say	1	9
Religion:		
Christian	6	54.5
Muslim	3	27.2
Spiritual	1	9
Prefer not to say	1	9
First language:		
English	6	54.5
Arabic	3	27.2
Igbo	1	9
Other	1	9
Prefer not to say	1	9
Disability:		
Yes	1	9
No	9	81.8
Prefer not to say	1	9
Please state your main occupation:		
Homemaker	2	18.1
Clerical and intermediate occupations	1	9
Modern professional occupations	3	27.2
Senior managers or administrators	2	18.1
Unemployed (including student, retired)	1	9
Other: Business owner	1	9
Prefer not to say	1	9

### Stages and nature of involvement

PPI contributors were involved at multiple stages

#### Funding and design

Prior to obtaining funding three development workshops were conducted with research delivery staff (*n* = 4) and women from underrepresented groups including Black women (*n* = 4). The workshops highlighted the need to focus research efforts specifically on Black women rather than broader ethnic groupings.

#### Data interpretation

During PPI consultation meetings PPI representatives shared personal experiences and perspectives in relation to study findings which enabled sense-checking and contextualising findings from the systematic review and qualitative focus groups.

#### Co-design

PPI representatives attended consensus workshops in which they contributed towards the development and refinement of the Principles of Engagement guidance.

#### Dissemination

PPI representatives have been involved in dissemination by providing testimonials, supporting accessible communication of findings, presenting at conferences, and co-authoring papers.

Involvement shifted over time from consultation toward co-interpretation and co-design, particularly during analysis and output development. For example, PPI members went from giving feedback on the findings of the PROMISE systematic review, to contributing towards the ‘Bridges and Barriers’ analogy to conceptualise the challenges that Black women experience when ‘crossing over’ to the world of research [27].

### Practicalities of involvement

Meetings were held online to reduce geographical and logistical barriers, scheduled to avoid school drop-off and pick-up times, and contributors were reimbursed following NIHR guidance. PPI activities (totalling 35 h) took place across the duration of the PROMISE study (12 months) and included consultation meetings, consensus workshops, surveys, written feedback exercises, and optional testimonials. Multiple approaches were used to accommodate different communication preferences and caring responsibilities whilst ensuring ongoing opportunities for reflection and contribution.

## Results

Drawing upon the GRIPP2 long-form framework (see Supplementary Material 1 for the full framework), we present the results of PPI in PROMISE across outcomes, impacts, contextual and process factors, and emergent conceptual learning. This relates to Sect.  7 (7a-7g) of the GRIPP2 long-form framework and for clarity, these are presented in Table 2, which provides an overview of our key learning mapped against core elements of the GRIPP2 framework. Rather than reporting activities alone, we focused on what changed because of PPI, how those changes occurred, and what this revealed about conducting inclusive PPI with communities experiencing structural inequities. PPI co-authors present their key learning from this reflexive article in Table 3 to ensure these messages maintain integrity.

**Table 2:** Lessons learned in PROMISE mapped against the GRIPP2 framework

GRIPP2 domain	What we did in PROMISE	What we learnt about effective PPI
Aims & design	Embedded PPI from funding stage; partnered with a community engagement organisation	Inclusive PPI requires early investment and specialist expertise; it cannot be retrofitted without loss of trust or influence
People involved	Purposively recruited Black women with lived maternity experience via focus groups and collaborated with trusted community groups	. Representation is relational, reflexive and demographic; ongoing attention to positionality and power was necessary throughout the study, when Black PPI members were working with researchers who were White and Asian.
Stages of involvement	Involved PPI contributors across design, interpretation, and co-design	PPI has greatest impact during interpretation and sense-making, where lived experience challenges assumptions
Nature of involvement	Moved from consultation to co-interpretation and co-design	Shared ownership develops over time and requires researchers to welcome uncertainty and critique
Context	Worked with trusted third-sector organisations; offered flexible engagement	Trust is context-specific and must be built and sustained in relation to the topic area.
Process	Prioritised flexibility, respectful facilitation, and clear communication	Engagement quality is central to inclusion and retention, particularly in emotionally sensitive research
	Were clear and transparent that, as a non-Black research team we wanted to hear the voices of Black women	Inclusive PPI requires explicit, visible commitment (e.g. targeted recruitment, culturally safe spaces, financial recognition), not passive openness
Impact	PPI shaped study focus, design, analysis, and outputs	Impact is often conceptual and relational, and difficult to capture through standard metrics
Reflection	Created space for contributors to reflect on involvement	Reflexivity is an ethical practice, not an optional add-on

### 7a. Outcomes of PPI: What PPI changed in the study

PPI influenced PROMISE at multiple stages, producing tangible changes to the study’s focus, methods, analysis, and outputs. The learnings from this work are particularly significant to methodology research, where decisions about how studies are designed, conducted, and interpreted have implications for the inclusivity and validity of future research.

#### Refinement of study focus

Early PPI input reinforced the need to focus explicitly on Black women rather than grouping experiences under broad “minoritised ethnic” categories. Contributors articulated how such aggregation risks erasing distinct histories of mistrust, differential treatment in maternity care, and culturally specific communication barriers. As a result, PROMISE adopted a specific focus on Black women, which shaped recruitment strategies, analysis, and dissemination plans.

#### Recruitment and engagement strategies

PPI contributors and community partners informed the framing of study materials, including the use of culturally resonant language and imagery, and the explicit naming of Black women as the population of interest, and importantly explaining why this population was the focus. This was intended not only to increase relevance, but to signal intentionality and respect, helping to counter assumptions that research engagement is tokenistic or extractive.

#### Interpretation and sense-making of findings

PPI contributors were particularly influential during data interpretation. When presented with emerging findings from focus groups and the systematic review, contributors contextualised discrepancies between Research Delivery Teams’ assumptions and Black women’s accounts of engagement. This reframing shifted interpretation away from individual-level explanations (e.g. “lack of awareness” or “reluctance”) toward structural factors including prior harm, communication styles, power imbalances, and emotional safety. In addition, PPI were instrumental in the development of a ‘Bridges and Barriers’ analogy, which was used to illustrate how various factors, identified in a systematic review, can facilitate or prevent Black women from ‘crossing over’ to the world of research. This provided a clear framework to guide culturally sensitive implementation for researchers, healthcare professionals, and policymakers.

#### Co-design of outputs

PPI contributors played a central role in refining the Principles of Engagement guidance through consensus workshops. Their input shaped both the content and tone of recommendations, ensuring they prioritised relational behaviours (e.g. respect, listening, trust-building) rather than technical fixes alone (e.g. translation or written materials). Initially, the principles of engagement were aimed at Research Delivery Teams, but during PPI consultations, it became clear that that these principles could also act as ‘promises’ to Black women to help overcome historical mistrust in research, openly acknowledging that Black women are important contributors in research and clearly outlining how they should expect to be treated in research.

### 7b. Impacts of PPI: Who and what was affected

#### Impact on the research

PPI strengthened methodological rigour by improving the cultural validity of interpretations (e.g. challenging deficit-based readings of hesitation or non-participation by reframing these as rational responses to prior experiences of exclusion and mistrust), questioning unexamined assumptions held by researchers (e.g. highlighting the importance of timing and tone), and ensuring outputs were grounded in lived experience rather than abstract principles (e.g. ensuring our outputs reflected the experiences of Black women, as opposed to the scientific research process and findings). The most significant impacts occurred during analysis and synthesis, rather than solely at the design stage, highlighting the value of sustained PPI user involvement.

#### Impact on contributors

Contributor feedback indicated that involvement was experienced as meaningful and respectful, particularly where experiential knowledge was treated as expertise, disagreements with researchers were welcomed constructively, rather than managed away, and there were visible links between contribution and change. Contributors described the value of influencing research that addressed long-standing inequities in maternal health, and of seeing their perspectives reflected in emerging outputs. Group meetings were also identified as impactful, providing a supportive space to share experiences, discuss findings, and connect with other women who held similar concerns and motivations. This collective setting reinforced contributors’ sense that their involvement extended beyond their individual input to a more overarching shared purpose and solidarity with other Black women.

**Box 1:** PPI feedback

*“I have loved being part of The PROMISE study*,* throughout the study we have been listened to and treated respectfully by all the researchers creating a space where we felt seen*,* heard*,* and respected*,* something that is often missing in maternal healthcare experiences. I attended all the workshops and consensus sessions and it was very comfortable to share ideas and perspectives. The PROMISE study is very important*,* and I have learned so much through taking part in it. Having a Black woman like myself involved helps ensure that research is conducted with greater understanding*,* care*,* and cultural awareness. I believe this will shape how research is done in the future and*,* ultimately*,* lead to better outcomes for pregnant women”.” – PPI contributor 1*	
*“Maternal health research for Black Women is very important because so many of our experiences are still being overlooked or dismissed. We know that Black Women face higher risks during pregnancy and childbirth*,* and a lot of that comes down to not being listened to or supported properly. Research like PROMISE will definitely help give our lived experiences a voice and highlight what actually needs to change. […] I wanted to take part in the PROMISE study because it felt different – it stated it was a maternal health research about Black Women. After the first session*,* I discovered it wasn’t just about numbers or data*,* but about lived experiences. I felt like this was a chance to lend my voice and be part of something that could make a real difference for other Black women. […] PROMISE is important because it’s about real change for Black Women. My involvement matters because every voice counts*,* and when we speak together*,* the change we want to see starts to happen. Being part of PROMISE feels like a way of standing up for the future of maternal health for Black Women.” – PPI contributor 2*	

#### Wider and potential impacts

At a broader level, PROMISE PPI contributed to the development of practical guidance for Research Delivery Teams and created capacity for future collaborations between researchers and community organisations. While longer-term impacts are yet to be evaluated, the process established relationships and practices that may support more equitable research engagement beyond this study.

### 7c. Context of PPI: What enabled or constrained involvement

Several contextual factors were critical in enabling inclusive PPI in PROMISE, whilst challenges were also apparent.

#### Enabling contextual factors

- Dedicated funding for PPI and community engagement from the outset.
- Partnership with a trusted community engagement organisation (Egality Health).
- Institutional expertise in PPI among the research team.
- Online delivery of sessions, which reduced geographical, time, and childcare barriers.
- Interactive and accessible consultation meetings.
- Awareness of cultural and historical experiences of mistrust.

#### Constraining contextual factors

- Limited project timescales constrained opportunities for deeper relationship-building.
- Structural inequities within research funding and delivery systems shaped what could realistically be achieved.
- Need for time and sensitivity given traumatic pregnancy/birth experiences.
- Emotional intensity of the topic required careful facilitation and support, which is not routinely resourced or acknowledged.
- The lack of representation of Black women within the research team was explicitly acknowledged in PPI discussions, with researchers using these spaces to reflect on positionality, invite challenge, and adapt decision-making in response to PPI input throughout the study.
- Online engagement may have excluded women with limited digital access, lower digital confidence, or restricted access to private devices and internet connectivity.

These contextual factors highlight that inclusive PPI is highly sensitive to structural conditions and cannot be separated from wider research system incentives and constraints.

### 7d. Process of PPI: How involvement worked in practice

Process quality was central to sustaining engagement and enabling meaningful contribution. Process features that supported good PPI included flexible modes of involvement; careful attention to the timing of meetings around caring responsibilities; and proactive efforts to remove practical barriers, such as offering online meetings and explicitly welcoming babies and breastfeeding. Clear, transparent communication about expectations, roles, and boundaries was paired with being explicit about the intention to involve Black women in the research, signalling respect and avoiding perceptions of tokenism. Respectful facilitation was critical, with an emphasis on active listening, cultural sensitivity, and acknowledging both power dynamics and the emotional labour associated with discussing potentially traumatic pregnancy and birth experiences. Research findings were also often translated into short vignette-based scenarios, grounding the evidence in real-life situations and supporting reflection, discussion, and meaningful engagement for a non-academic audience.

Financial recognition of contributors’ time (e.g. vouchers) and engagement through trusted community organisations enabled access to established networks and “borrowed trust,” further supporting inclusive participation. Together, these practices highlighted that meaningful PPI requires not only flexibility, but intentional, visible efforts to create emotionally safe, culturally responsive, and accessible spaces for involvement.

Shared consensus workshops brought together PPI contributors and Research Delivery Team members, creating a mixed space that required careful facilitation. This was supported by PPI contributors’ prior involvement in the study; they were familiar with the research team and research aims, understood how PPI lived experience had shaped the work, and were confident that their perspectives were valued. The research team remained attentive to the power dynamics of the consensus workshops and aimed for a balance of perspectives from all stakeholder groups on each of the PROMISE principles. As such, PPI contributors were at times actively invited to speak with alternative routes for communication and contribution (including written feedback) offered throughout.

Despite these efforts, PPI contributors reflected that power dynamics in these spaces remained weighted towards Research Delivery Teams. This highlighted the limits of relational familiarity alone. During reflective discussions, contributors and researchers identified several approaches that were not implemented within PROMISE but may strengthen future PPI practice, including dedicated contributor-only spaces, staged engagement processes, and interactive co-design approaches, such as dot voting and word clouds. Some suggested that a larger PPI group may have further broadened perspectives, and that alternative communication approaches (e.g. messaging platforms such as WhatsApp rather than email) may have improved attendance.

PROMISE demonstrated that how PPI is conducted matters as much as when or with whom. Contributors were most engaged when processes felt relational rather than transactional, and when their input was visibly taken seriously even where it complicated analysis or challenged researcher assumptions. These reflections highlighted that inclusive PPI requires ongoing adaptation to the specific needs of a study.

### 7ei–7eii. Emergent conceptual and theoretical learning

PROMISE contributes to emerging conceptual understanding of PPI in three ways:

1. *Ethnically specific PPI as methodologically distinct. *The study illustrates how PPI with Black women requires attention to trust and historical context affecting Black women specifically.
2. *Interpretive labour as a core to PPI value. *PPI impact was strongest during analytical sense-making, suggesting that involvement in interpretation merits greater emphasis in PPI design and reporting.
3. *Trust as a relational and transferable mechanism. *Trust was not static or assumed, but actively built through partnership, transparency, and respect. This was reinforced through engagement with respected community figures (e.g. Black Mama’s Birth Village) whose credibility and authenticity helped create a sense of interest in participation, safety and legitimacy, drawing others into conversations and modelling confidence in the research process.
4. *Inclusive PPI as a driver of methodological innovation. *The study highlights that involving Black women in methodology research can fundamentally reshape research processes, challenging assumptions and improving how studies are designed and interpreted.

These insights extend existing PPI frameworks by foregrounding relational and contextual mechanisms rather than procedural checklists.

### 7 f. Measurement of PPI impact

Formal quantitative measurement of PPI impact was not undertaken. Impact was primarily evidenced through qualitative indicators such as, documented changes to study focus and outputs, researcher reflexive notes and contributor feedback and testimonials. While consistent with much of the PPI literature, this highlights ongoing challenges in evidencing impact in ways that remain meaningful to contributors while also credible within academic reporting standards.

### 7 g. Economic considerations related to PPI

A formal economic evaluation of PPI was outside the scope of this study. However, inclusive PPI required dedicated funding for community engagement expertise, reimbursement of contributors’ time and expenses, additional researcher time for planning, facilitation, and reflection. These costs should be understood as integral to research quality rather than discretionary add-ons. Failure to resource PPI adequately risks superficial involvement and inequitable practice. In addition, there is a need to consider supporting PPI prior to receiving funding; with many universities cutting internal research budgets this is a growing challenge and area of concern for the development of future research proposals.

### PPI informed key lessons

The following lessons were co-developed with Black women PPI contributors involved in the PROMISE study and also our PPI co-authors. The lessons reflect their experiences of meaningful involvement to support future PPI research with Black women.

**Table 3:** Lessons for conducting inclusive PPI *with* Black women, *from* Black women

Key lesson	Why this matters	Implications for researchers	Implications for PPI contributors
Understand the historical and cultural barriers shaping participation	Many Black women approach research cautiously due to experiences of being overlooked, misunderstood, or harmed within healthcare and research systems. These experiences shape trust, willingness to participate, and how research interactions are interpreted.	Researchers should develop cultural awareness and understand how racism, mistrust, and previous healthcare experiences may influence engagement. Research processes should be adapted to the communities they aim to involve, rather than expecting communities to adapt to research systems.	Research can often be emotionally and physical difficult for PPI contributors. They should feel able to discuss how past experiences influence their trust in research and what would help involvement feel safe, respectful, and meaningful.
Actively listen and approach traumatic experiences with sensitivity and care	Discussions about pregnancy, birth, racism, and mistrust can be emotionally difficult. There is a breakdown in trust between Black women and healthcare, as well as other related systems such as medical research. Many Black women fear not being listened to or having concerns dismissed. Maternity research can exacerbate these concerns as women worry about putting not only themselves, but also their baby, ‘at-risk’.	Researchers should create emotionally safe spaces, allow time for reflection, and approach traumatic experiences with empathy and compassion. Active listening and responsiveness are central to rebuilding trust.	Contributors should feel respected, heard, and able to share experiences without pressure. Meaningful collaboration develops when contributors can see that their feedback is genuinely valued and acted upon.
Remove practical barriers and build flexibility into involvement	Caring responsibilities, work, geography, childcare, and financial pressures can all limit participation. Flexible approaches make involvement more accessible and inclusive.	Researchers should design PPI around people’s lives rather than academic convenience. This includes offering online and hybrid meetings, flexible ways to contribute, reimbursement, accessible materials, and scheduling that accommodates parents and carers.	Contributors should be able to identify what would make involvement easier and more realistic for them, including requesting different formats, written feedback options, camera-off participation, or additional time to reflect before responding.
Be clear, honest, and explicit about wanting Black women to be involved	Generic language about “diversity” or “underserved groups” can feel vague or tokenistic. Explicitly naming Black women signals intentionality, respect, and recognition that their experiences are distinct.	Researchers should be transparent about why Black women’s involvement is important, how their input will shape the research, and the limits and expectations of involvement. Clear communication about confidentiality, withdrawal, and decision-making is essential.	Contributors should feel able to ask why they are being invited to participate and how their voices will influence the research. They should have input into whether study framing feels respectful and meaningful.
Trust must be built before meaningful involvement can occur	Black women may enter research spaces carrying experiences of mistrust, dismissal, or harm within healthcare systems. Trust cannot be assumed simply because someone agrees to participate. In PROMISE, trust developed through respectful communication, transparency, consistency, and acknowledgement of privilege and positionality by researchers.	Researchers should invest time in relationship-building and avoid prioritising outputs or consensus over meaningful engagement. Trust develops when contributors can question, challenge, and see that their input leads to visible changes in the research.	Contributors should not feel pressured to agree quickly or communicate in academic ways. Questions, concerns, and hesitation are valuable and form part of understanding what meaningful involvement requires.
Lived experience should be recognised as expertise, particularly during interpretation	One of the most valuable aspects of PROMISE was working towards sharing research power equitably with Black women, including them in sense-checking and interpreting findings. In addition, during the focus groups some Black women helped interpret some of the research concepts (e.g. giving consent) to others using their native language.	Researchers should involve PPI contributors across all stages of the research, including in analysis and interpretation, not only at recruitment or dissemination stages. Researchers must be willing to share research power, have assumptions challenged and treat disagreement as analytically valuable.	Contributors should feel confident that their role extends beyond sharing experiences to shaping data collection, interpretation, challenging assumptions, and influencing the meaning drawn from the research.

## Discussion

Using the GRIPP2 long-form framework as a reflective lens, this paper has examined how PPI functioned within the PROMISE study and what this revealed about conducting inclusive, ethnically specific PPI in maternal methodology research. Rather than reiterating activities or outcomes, the discussion focuses on the conceptual, methodological and practical implications of these findings, and the conditions required for meaningful involvement. These findings are particularly important in the context of methodology research, where PPI remains limited despite its potential to shape how research is designed and delivered [7, 8], and therefore the likelihood that innovations are fit for purpose and reflect real life experiences. Methodology research can directly influence the assumptions, processes, and tools that underpin future research. As such, the absence of inclusive PPI in methodology work risks embedding inequities within research systems themselves.

Despite growing emphasis on PPI, involvement practices often remain demographically limited and risk reinforcing exclusion where relational and contextual considerations are marginalised [3, 24]. This study highlights how attention to power, trust, context, and long-term relationship-building can enable more meaningful involvement with Black women and enhance the interpretive robustness of methodological research. The focus on Black women, as opposed to more generic “minoritised” groups, created space to engage directly with the structural/historical dynamics shaping participation in maternity research for this group.

The study also illustrates that good PPI is shaped by context and process. Early partnership with a community engagement organisation, and prior PPI experience within the research team were all enabling conditions. These contextual supports created space for flexibility, responsiveness, and reflection. Process factors, including respectful facilitation, transparency about decisions, and visible links between contributors’ input and changes to the research, were critical in sustaining engagement. Productive involvement required researchers to tolerate discomfort, contradiction, and critique, rather than seeking reassurance or consensus. This approach is reflected in the co-developed lessons for conducting inclusion PPI with Black women reported in Table 3. These lessons extend beyond procedural recommendations to highlight the relational, emotional and structural aspects of involvement that contributors identified as key to meaningful participation. Positioning these lessons explicitly within the voices and reflections of Black women helped to ensure that practical guidance remains grounded in lived experiences rather than researcher assumptions alone.

The value of PPI was most evident at the stages of interpretation and synthesis. While involvement remains predominantly concentrated at the design phase of research, contributors in PROMISE acted as interpretive partners, challenging researcher assumptions and reframing explanations of research participation. This shifted the analytical focus away from potential deficit-based interpretations and towards structural mistrust, prior harm, and emotional safety. Although evidence suggests that such interpretive involvement can enhance analytic quality, it remains under-reported and insufficiently resourced within prevailing PPI guidance and practice [3].

Despite its strengths, the PROMISE approach was resource-intensive and relied on conditions that are not routinely supported within research. Meaningful and inclusive PPI required sustained funding, specialist expertise, and considerable researcher time, raising concerns about its scalability and sustainability in constrained funding environments. This highlights a broader structural tension: while inclusive PPI is widely endorsed, it is often insufficiently resourced, resulting in disproportionate practical and emotional demands falling on a small number of contributors and researchers [25, 29]. Importantly, contributors emphasised that targeted efforts to recruit and involve Black women should never be treated as optional, but rather as a core component of rigorous and equitable research practice. Failure to resource and prioritise such efforts risks reinforcing the very inequities that inclusive PPI seeks to address. The concept of ‘emotional labour’, in this case discussing experiences of racism and mistrust in maternity care, is largely unrecognised in research processes [29], raising ethical questions about reciprocity, care, and whether reimbursement alone is an adequate response to the nature of contributors’ involvement. Furthermore, as in many PPI initiatives, a relatively small group of contributors was engaged, raising questions about how far their perspectives reflect wider community experiences. Indeed, although contributors reflected diversity across Black ethnicities, migration histories, occupations, and educational backgrounds, several participants were well educated and had previous experience engaging with research. As such, perspectives from Black women who are less connected to research, have lower levels of formal education, experience greater socioeconomic disadvantage, or face additional barriers to participation may be underrepresented. This reflects a wider challenge within PPI and highlights the ongoing need for alternative engagement approaches that reach individuals who remain less visible within research structures. Although PROMISE sought to mitigate this through community partnerships, this remains an ongoing challenge in work with communities experiencing structural inequities .

While representation within research teams is often highlighted as important for trust and engagement, PROMISE demonstrates that meaningful PPI with Black women was possible despite the research team not being racially concordant with contributors. Trust was built through transparent acknowledgement of positionality, partnership with trusted community organisations, continuity of relationships, and willingness to share power in the research process. This finding aligns with evidence suggesting that while racial concordance can support trust and communication, culturally humble, reflexive, and community-embedded practices can also foster meaningful engagement in racially non-concordant research teams [27, 28, 30].

The impact and implementation of PPI represented a further challenge. While contributors had genuine influence, not all suggestions could be implemented within a tightly resourced piece of funded research. This required transparent communication about the limits of involvement. Capturing the impact of PPI also remained challenging. As with much of the PPI literature, the most significant impacts in PROMISE were conceptual and interpretive rather than readily quantifiable (2, 3. Whilst GRIPP2 supported structural reflection, current approaches tend to prioritise measurable outcomes in PPI evaluation over relation and experiential dimensions of involvement. As such, there remains a need for further development of contributor-defined indicators of meaningful involvement. Complementary frameworks, such as the Patient-Focused Medicines Development (PFMD) Patient Engagement Quality Guidance, offer a useful extension by foregrounding contributor-defined indicators of meaningful engagement, including respect, support, transparency, and shared purpose [31]. However, these approaches remain under-integrated within mainstream PPI evaluation. There remains a need for further development and uptake of contributor-defined indicators that capture the quality and meaning of involvement, alongside its more tangible impacts.

## Conclusion

The PROMISE study provides empirical insight into how PPI with Black women can be enacted within maternity methodology research. Through sustained partnership with Black women and the use of GRIPP2 as a reflective lens, this reflection piece highlights the importance of addressing historical mistrust and recognising lived experience as central to interpreting findings and shaping methodological practice. The co-developed lessons provided here offer a practical foundation for moving beyond generic inclusion towards more culturally responsive, relational approaches to PPI across healthcare and methodology research. Using GRIPP2 enabled a shift from descriptive reporting to critical reflection on how PPI operates in practice, including its tensions and limitations, and the conditions needed to support meaningful, equitable involvement.

## Supplementary Information

Below is the link to the electronic supplementary material.

Supplementary Material 1

## Data Availability

No datasets were generated or analysed during the current study.

## Abbreviations

GRIPP2: Guidance for Reporting Involvement of Patients and the Public (version 2)
INCLUDE: NIHR Innovations in Clinical Trial Design and Delivery for the Under-served
NIHR: National Institute for Health and Care Research
PPI: Patient and Public Involvement
PROMISE: Principles of Engagement: Developing Methods to Increase Representation of Black Mothers in Maternal and Neonatal Healthcare Research
